# Training Preceptors of Obstetrics-Gynecology Residents through the One-minute Preceptor Model

**DOI:** 10.1055/s-0041-1735230

**Published:** 2021-09-21

**Authors:** Michelle Araújo Machado, Elaine Lira Medeiros

**Affiliations:** 1 Department of Gynecology, Universidade Federal do Rio Grande do Norte, Natal, Rio Grande do Norte, RN, Brazil

**Keywords:** preceptorship, training courses, internship and residency, formative feedback, medical education, preceptoria, cursos de capacitação, internato e residência, *feedback* formativo, educação médica

## Abstract

**Objective** To analyze the effect of the One-minute Preceptor model for preceptors who work at the emergency department of a maternity teaching hospital.

**Methods** A quantitative intervention study conducted with Obstetrics and Gynecology residency preceptors at a maternity teaching hospital in northeastern Brazil. Three stages were performed: 1) a preintervention survey with the residents; 2) planning and execution of a pedagogical training course for the preceptors, which involved a lecture and a dramatization about the One-Minute Preceptor model; and 3) thirty days after the intervention, the residents answered another survey about the model and its repercussions and advantages.

**Results** The preintervention assessment with the residents showed that 91.7% agreed that there were discrepancies regarding the teaching model among the preceptors. After the training, all preceptors agreed that the model engages the student in the decision-making process, and that they would apply it to their routine. The postintervention results showed that 95.8% agreed that the model is more inviting than traditional teaching approaches. There was a perception of improvement in learning among 70.9% of the residents. In addition, the present study found a significant change in feedback before and after implementing the model, from 20.8% to 66.7%.

**Conclusion** The training course of preceptors in the One-Minute Preceptor model proved to be efficient in providing formative feedback to residents in the emergency department of a maternity school. Further studies are needed to assess the consolidation of the methodology in the long term.

## Resumo

**Objetivo** Analisar o efeito de uma capacitação no modelo *One-Minute Preceptor* para preceptores que atuam no setor de urgência de uma maternidade escola.

**Métodos** Estudo de intervenção, quantitativo, realizado com preceptores de residência em Ginecologia e Obstetrícia de uma maternidade escola do Nordeste brasileiro. Três etapas foram realizadas: 1) questionário pré-intervenção com os residentes; 2) planejamento e execução de um curso de capacitação pedagógica para os preceptores, que envolveu uma aula expositiva dialogada e dramatização sobre o modelo *One-Minute Preceptor* ; e 3) trinta dias após a intervenção, os residentes responderam a outro questionário sobre a aplicação, a repercussão e as vantagens do modelo.

**Resultados** Na avaliação de ensino dos residentes pré-intervenção, os resultados mostraram que 91,7% concordaram que existiam divergências quanto a metodologia de ensino entre os preceptores. Após a capacitação, todos os preceptores concordaram que o método envolve o aluno no processo de tomada de decisão, e que aplicariam o método na sua rotina no setor de urgência. Os resultados pós-intervenção mostraram que 95,8% concordam que o modelo é mais atratente do que as abordagens de ensino tradicionais. Para 70,9% dos residentes, houve uma percepção de melhora da aprendizagem; além disso, observou-se uma mudança siginificativa do recebimento de *feedback* antes e após a implantação do modelo, de 20,8% para 66,7%.

**Conclusão** A capacitação de preceptores no modelo *One Minute Preceptor* mostrou-se eficiente no fornecimento de *feedback* formativo aos residentes no setor de urgência de uma maternidade escola. Novos estudos serão necessários para avaliar a consolidação da metodologia em longo prazo.

## Introduction

Residency is considered the gold standard of specialization programs, and its purpose is to improve the professional competency acquired during the undergraduate medicine course. In addition to teaching knowledge and skills, it also enhances the students' relationship attributes, postures, and attitudes needed to achieve medical professionalism. The resident learns through a wide range of techniques, thus achieving professional quality in its fullness. 1 2 3 4

The figure of the preceptor has been a part of medical education for many years. And if medical residency has been documented since 1889, learning from more experienced doctors has been recognized since the dawn of medicine, 4 for they provide guidance, support, teaching, and knowledge of their experiences. 5

Despite having an extremely important role in the training of medical students, residency preceptors have little experience with pedagogical training. Without an adequate follow-up from them, students are left loose and unassisted; thus, exposed to poor practices that will have a negative influence on their education. 6 When exercising preceptorship, the doctor must master not only clinical knowledge, but they must be able to transform field experience into teaching, in addition to conveying pedagogical knowledge. 7

Regarding the pedagogical knowledge needed to train a preceptor, gaps are evident. 8 9 10 On the other hand, higher-education institutions have been pressured to change the way these professionals are qualified, and are currently meeting the demand to form instructors who can transform realities. The guidelines for medical syllabi corroborate this idea of critical and reflective teaching through the implementation of methodologies that encourage students to learn and to reflect upon social reality. 11

Active learning, which emerged in the 1960s, is a teaching-learning strategy based on problematization to reach and motivate the student through keen involvement in their training, while the preceptor acts only as a facilitator. Its use can be the solution to develop student autonomy, forming creative, reflective, and independent professionals. 12 13

In 1992, researchers from the Department of Family Medicine at the University of Washington 14 introduced, in outpatient clinics, a new model of preceptorship which could also be used in emergency rooms and wards. The model's theory provides the preceptor with the ability to convey valuable medical information to their students in a fast and effective manner, and to comprehend the particularities of each student. Called One-Minute Preceptor (OMP), this medical teaching model was developed to be used when a student or resident, after assessing a clinical case, requests the assistance of their preceptor to solve problems. It consists of five fundamentals steps (microskills): 1) get a commitment: getting a commitment from the learner about what is going on with the patient, asking them to state the probable diagnoses; 2) probe for supporting evidence: probe the learner for their underlying reasoning, encouraging them to state aspects regarding the differential diagnosis; 3) teach general rules: based on the answers to steps 1 and 2, teach general principles about the clinical case; 4) reinforce what was done right: provide positive and formative feedback to the learner; and 5) correct mistakes: correct errors for future performance. 14

Some authors 15 suggest the OMP model as a framework for an efficient and precise preceptorship, which enables learning in limited time due to patient-care demands. Considering the relevance of this issue, the present study aimed to analyze the effect of the OMP model on preceptors who work at the emergency department of a maternity teaching hospital in northeastern Brazil.

## Methods

The present was a cross-sectional study associated with the Obstetrics and Gynecology (OBG-GYN) residency program at a maternity teaching hospital in the city of Natal, Brazil. Data collection occurred between August and November 2019. Of the 28 eligible preceptors who worked at the Emergency Department, 15 volunteered to participate in a training course. As for the residents, 24 out of 28 answered surveys before and after the educational intervention alongside with the preceptors.

Initially, a face-to-face survey was applied to OB-GYN residents to identify their sociodemographic characteristics, the year of residence they were attending, questions regarding the teaching methods used by the preceptors, the feedback, and their impressions on the quality of teaching (questionnaire A).

The training course was held in October 2019, at the maternity auditorium; it lasted 3 hours, and was taught during the night shift. A badge containing the OMP steps on the back was given to each tutor.

First, there was a seminar on the OMP addressing the following topics: 1) difficulties encountered while exercising preceptorship; 2) the need for educational training courses; 3) division between teaching and practice in a high-flow environment; 4) professional competency versus pedagogical competency; 5) comparison between the traditional and active methodologies; and 6) presentation of the OMP, step by step. Questions and further discussions were held at the end of the lecture.

Later, role-playing was performed by a fellow and a resident; both had previously been trained by the researcher. The case study was about severe preeclampsia, and it was managed in two contrasting ways: the first one, by an “inadequate” tutor, using the traditional approach, without considering the student's autonomy, and the second, using the OMP. After the role-play, some preceptors were invited to reproduce case discussions using the OMP model.

In the end, a satisfaction survey (questionnaire B) was applied to assess the duration of the course, the teaching method, the applicability of the OMP model, and the degree of comfort in providing feedback; the survey also contained a blank space for suggestions and critiques. Thirty days after the training, another survey (questionnaire C) was answered, in person, by the residents about their perceptions of the OMP and its applicability during shifts.

At first, a database was created to organize the data. Next, tables of absolute and relative frequencies were built to characterize the residents regarding their sociodemographic characteristics and to analyze questionnaires A, B, and C, whose alternatives were planned according to the Likert scale. 16 For the data analysis, we used the Microsoft Office Excel (Microsoft Corp., Redmond, WA, US) software, version 2016, and the McNamer test. The second one was used for the quantitative analysis of the paired variables to verify the association between two categorical variables (receiving feedback before and after). A significance level of 5% was adopted for all tests. The statistical analysis was performed using the Statistical Package for the Social Sciences (IBM SPSS Statistics for Windows, IBM Corp., Armonk, NY, US) software, version 21.

The study was approved by the institutional Ethics in Research Committee under protocol number 3.483.115, CAAE 17211319.6.0000.5292, in August 2019. A consent form was provided to the preceptors and residents who took part in the study.

## Results

The results of the survey results are presented in three subtopics: the first one contains data from the residents before the intervention; the second one contains information from the preceptors; and the third contains data from residents after the training with the preceptors.

### Preintervention: The Resident's Perspective on the Teaching-Learning Process

The study identified that 91.7% ( *n* = 22) of the residents completely or partially agreed that there were differences in the methodology used by the preceptors: 54.2% ( *n* = 13) agreed completely, and 37.5% ( *n* = 9), partially. We noted that 62.5% ( *n* = 15) of the residents partially agreed that the central figure in the teaching-learning process was the preceptor-patient relationship. Regarding the stimulation of clinical reasoning, 71% ( *n* = 17) of the residents felt they were partially (41.8%) or totally (29.2%) active in the teaching-learning process. On the other hand, 75% ( *n* = 18) disagreed, totally (46%) or partially (29%), about receiving feedback. However, 70.8% ( *n* = 17) rated the quality of the teaching as good.

### Intervention: Training Preceptors in the OMP Model

The preceptors were predominantly female (93.3%) and associated with the state-owned Empresa Brasileira de Serviços Hospitalares (Brazilian Hospital Services Company, EBSERH, in Portuguese) (80%). They joined the training on the OMP model and subsequently evaluated the intervention. They indicated that the duration of the course was adequate, the presentation was satisfactory, and the role-playing was easy to understand.

The data showed that 86.7% ( *n* = 13) of the preceptors agreed that the OMP model involves the student in the decision-making process, and 93.3% ( *n* = 14) agreed, totally (60%) or partially (33.3%), that the OMP would streamline the service flow. All participants indicated that they would start applying the model to their routines.

As for the provision of feedback, after the training, 86.7% ( *n* = 13) of the preceptors answered that they felt comfortable, and only 13.3% ( *n* = 2) reported they felt uncomfortable doing it. We also observed that these preceptors did not provide feedback regularly due to the lack of training and did not undermine the residents' self-esteem.

At the end of the satisfaction survey, 7 (25%) preceptors suggested increasing the number of on-call staff to make the application of the model easier, the provision of ongoing education for them, a unification of their conduct, more time to discuss the cases, and issues regarding team recycling.

### Postintervention: The Perspective of the Residents after the Training

**Fig. 1:**
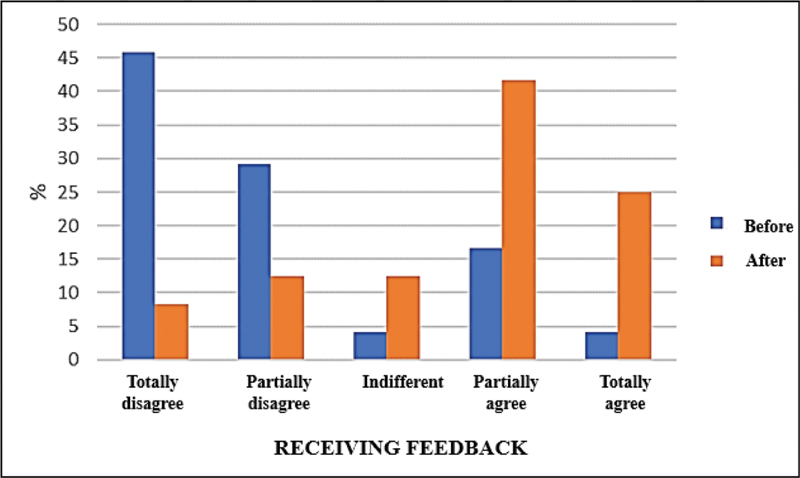
Perception of the residents on receiving feedback before and after the intervention.

After the training, 66.67% ( *n* = 16) of the residents agreed that the cases started to be discussed using the OMP model. Table 1 shows the result of the McNamer test used to assess the frequencies of two related samples. A statistically significant change was noted for the feedback received by the residents before and after the training: from 20.8% to 66.7% ( *p* = 0.021) ( Fig. 1 ).

**Table 1:** Perception of the residents on receiving feedback before and after the intervention using the McNamer test with a significance level of 5%

Receiving feedback	Before	After	Total	*p* -value
	n (%)	n (%)	n (%)	
Totally disagree	11 (45.83)	02 (8.33)	13 (27.07)	
Partially disagree	07 (29.17)	03 (12.50)	10 (20.83)	
Indifferent	01 (4.17)	03 (12.50)	04 (8.34)	0.021
Partially agree	04 (16.67)	10 (41.67)	14 (29.18)	
Totally agree	01 (4.16)	06 (25.00)	07 (14.58)	
Total	24 (100.00)	24 (100.00)	48 (100.00)	

A total of 33 (95.8%) residents also agreed that the OMP is a more interesting model than other traditional teaching methods, and only 4.2% ( *n* = 1), totally disagreed with the OMP. Finally, 70.9% of the residents completely agreed that there was an improvement in teaching and learning using the OMP model.

## Discussion

The present study showed that the preceptors who conducted the training on the OMP recognized that the model engages the student in the decision-making process and improves the service flow. All preceptors agreed to incorporate the model into their routines.

Likewise, an American study 17 conducted in 2018 offered workshops on the OMP to 294 staff physicians and 98 residents from 21 different medical specialties and subspecialties, at 16 different teaching hospitals, and found that 97% of participants would use the method frequently, even daily. It also established that the presentation and discussion of the model followed by role-playing, the same resources used in our study, can be effective to instruct the participants on the use of the OMP.

A recent article 18 from a Nursing college in Washington reported training 58 preceptors using methodologies focused on clinical teaching, and, among them, the OMP model. All participants rated the steps of the method as useful or very useful. Therefore, the authors pointed out that training preceptors in evidence-based and efficient strategies, such as the OMP, is feasible and effective.

A study 19 performed 2019 in the state of Pernambuco, Brazil, identified that, after training preceptors using the OMP, the implementation of the model for physiotherapy students was positively analyzed by both preceptors and students. The study also showed that preceptors trained using the method had improved regarding the provision of feedback and the teaching of critical reasoning to students.

The relevant data found by the present study was the significant increase in the provision of feedback after the intervention, thus contributing to improve the teaching-learning process. The residents considered feedback an important learning tool, and they valued teachers who knew how to provide it effectively.

A research 20 performed with interns and residents ( *n* = 3,471) of the outpatient clinics of five medical schools in Ontario, Canada, investigated the location characteristics and preceptor behaviors that contributed to the students' learning. It was then identified that 95% considered that providing feedback constructively is an essential attribute of the preceptor, taking second place among 36 other teaching skills listed, being surpassed only by the ability to be open to questions.

Nonetheless, a study 21 performed in Singapore, using a method similar to ours, involved 37 preceptors who were trained in workshops on OMP and 34 dentistry residents who evaluated them. The perceptions about clinical teaching were assessed before and after the training, and the authors concluded that the short duration of the training impaired the quantity and quality of the education. Thus, this study showed that a single workshop for faculty members did not substantially improve the perceptions of the residents regarding the quality or quantity of the education, which indicates that training should be ongoing.

## Conclusion

There was good acceptability of the OMP model by the preceptors. Moreover, the model was inserted in a standardized manner in the emergency work routine of the Obstetrics and Gynecology Department, as well as in the case discussions of the residents, who reported more autonomy provided by the model. In addition, there was an increase in the stimulus to clinical reasoning and to provide feedback, which were previously weak, and started to be provided regularly by the preceptors. Therefore, training preceptors on the OMP showed to be an important, viable, and effective strategy. Further training and investigations are needed to assess how this method develops in the long term.

## References

[1] Ministério da Educação Secretaria de Ensino Superior Residência Médica Latu Sensu Brasília, DF MEC 2012

[2] Sampaio S A A implantação da residência médica no hospital das clínicas: 40 anos de história Estud FUNDAP. 1984 1 4 32

[3] Botti S H O papel do preceptor na formação de médicos residentes: um estudo de residências em especialidades clínicas de um hospital de ensino [tese] Rio de Janeiro Escola Nacional de Saúde Pública Sergio Arouca 2009

[4] Martins L A Residência médica: estresse e crescimento São Paulo Casa do Psicólogo 2005

[5] Macedo C G Apresentação São Paulo Hucitec 1999 9 11

[6] Missaka H Ribeiro V M A preceptoria na formação médica: o que dizem os trabalhos nos congressos Brasileiros de educação médica 2007–2009 Rev Bras Educ Med 2011 35 03 303 310 10.1590/S0100-55022011000300002

[7] Ribeiro K R Prado M L A prática educativa dos preceptores nas residências em saúde: um estudo de reflexão Rev Gaúcha Enferm 2014 35 01 161 165 10.1590/1983-1447.2014.01.43731 24930287

[8] Rocha H C Ribeiro V B Curso de formação pedagógica para preceptores do internato médico Rev Bras Educ Med 2012 36 03 343 350 10.1590/S0100-55022012000500008

[9] Afonso D H Silveira L M Os desafios na formação de futuros preceptores no contexto de reorientação da educação médica Rev HUPE. 2012 11 01 82 86

[10] Silva V C Viana L O Santos C R A preceptoria na graduação em enfermagem: uma revisão integrativa da literatura Journal of Research: Fundamental Care Online 2013 5 05 20 28

[11] Ministério da Educação Conselho Nacional de Educação Câmara de Educação Superior Resolução CNE/CES No. 3, de 20 de junho de 2014 Institui diretrizes curriculares nacionais do curso de graduação em medicina. Diário Oficial da União Seç. 2014 1 8 11

[12] Araújo T A Vasconcelos A C Pessoa T R Forte F D Multiprofissionalidade e interprofissionalidade em uma residência hospitalar: o olhar de residentes e preceptores. Interface (Botucatu) 2017 21 62 601 613 10.1590/1807-57622016.0295

[13] Farias L M Manual do estágio curricular do curso de graduação em Enfermagem Brasília, DF Fundação de Ensino e Pesquisa em Ciências da Saúde/Escola Superior de Ciências da Saúde 2015

[14] Neher J O Gordon K C Meyer B Stevens N A five-step “microskills” model of clinical teaching J Am Board Fam Pract 1992 5 04 419 424 1496899

[15] Chemello D Manfroi W C Machado C L O papel do preceptor no ensino médico e o modelo preceptoria em um minuto Rev Bras Educ Med 2009 33 04 664 669 10.1590/S0100-55022009000400018

[16] Likert R A technique for the measurement of attitudes Arch Psychol 1932 22 140 1 55

[17] Servey J Wyrick K Teaching clinical precepting: a faculty development workshop using role-play MedEdPORTAL 2018 14 10718 10.15766/mep_2374-8265.10718 30800918 PMC6342365

[18] Fincham S J Smith T Purath J Implementation of an educational program to improve precepting skills J Am Assoc Nurse Pract 2019 33 04 331 337 10.1097/JXX.0000000000000326 31702606

[19] Pimentel C M Análise da implementação do modelo de ensino one minute preceptor na vivência da prática profissional de estudantes de fisioterapia de uma faculdade do Nordeste brasileiro [dissertação] Recife Faculdade Pernambucana de Saúde 2019

[20] Schultz K W Kirby J Delva D Godwin M Verma S Birtwhistle R Medical Students' and Residents' preferred site characteristics and preceptor behaviours for learning in the ambulatory setting: a cross-sectional survey BMC Med Educ 2004 4 12 10.1186/1472-6920-4-12 15298710 PMC514563

[21] Ong M M Yow M Tan J Compton S Perceived effectiveness of one-minute preceptor in microskills by residents in dental residency training at National Dental Centre Singapore Proc Singapore Healthcare. 2017 26 01 35 41 10.1177/2010105816666294

